# PretiMeth: precise prediction models for DNA methylation based on single methylation mark

**DOI:** 10.1186/s12864-020-6768-9

**Published:** 2020-05-15

**Authors:** Jianxiong Tang, Jianxiao Zou, Xiaoran Zhang, Mei Fan, Qi Tian, Shuyao Fu, Shihong Gao, Shicai Fan

**Affiliations:** 1 School of Automation Engineering, University of Electronic Science and Technology of China, Chengdu, 611731 China; 2 Department of Automation, Tsinghua University, Beijing, 100084 China; 3 Chengdu Women’s and Children’s Central Hospital, School of Medicine, University of Electronic Science and Technology of China, Chengdu, 611731 China; 4 Center for Informational Biology, University of Electronic Science and Technology of China, Chengdu, 611731 China

**Keywords:** DNA methylation, Single-locus modeling, Precise prediction, Logistic regression, TCGA, Differential methylation

## Abstract

**Background:**

The computational prediction of methylation levels at single CpG resolution is promising to explore the methylation levels of CpGs uncovered by existing array techniques, especially for the 450 K beadchip array data with huge reserves. General prediction models concentrate on improving the overall prediction accuracy for the bulk of CpG loci while neglecting whether each locus is precisely predicted. This leads to the limited application of the prediction results, especially when performing downstream analysis with high precision requirements.

**Results:**

Here we reported PretiMeth, a method for constructing precise prediction models for each single CpG locus. PretiMeth used a logistic regression algorithm to build a prediction model for each interested locus. Only one DNA methylation feature that shared the most similar methylation pattern with the CpG locus to be predicted was applied in the model. We found that PretiMeth outperformed other algorithms in the prediction accuracy, and kept robust across platforms and cell types. Furthermore, PretiMeth was applied to The Cancer Genome Atlas data (TCGA), the intensive analysis based on precise prediction results showed that several CpG loci and genes (differentially methylated between the tumor and normal samples) were worthy for further biological validation.

**Conclusion:**

The precise prediction of single CpG locus is important for both methylation array data expansion and downstream analysis of prediction results. PretiMeth achieved precise modeling for each CpG locus by using only one significant feature, which also suggested that our precise prediction models could be probably used for reference in the probe set design when the DNA methylation beadchip update. PretiMeth is provided as an open source tool via https://github.com/JxTang-bioinformatics/PretiMeth.

## Background

DNA methylation, as an important epigenetic modification, plays an important role in maintaining normal cell function, genetic imprinting, embryonic development and human tumorigenesis [1–6]. The investigation of the methylation landscape of the human genome and the aberrant methylation pattern resulting in different diseases is still a hot spot. DNA methylation is commonly detected by array or sequencing technique in cell lines or bulk tissue samples. DNA methylation data from genome-wide sequencing can provide more comprehensive methylation information, while the high cost of the current bisulfite sequencing platforms makes it impractical for the large-scale research [7]. The most common method for the characterization of DNA methylation in humans was the Illumina Infinium HumanMethylation 450 K BeadChip (450 K), which measured methylation at ~ 450,000 CpG loci throughout the genome [8]. In recent years, Illumina updated the 450 K array to Illumina InfiniumMethylation EPIC BeadChip (EPIC or 850 K), which provided more methylation information from the distal regulatory regions (eg, enhancer) and covered the CpG loci almost twice as much as 450 K array [9]. Till now, it is quite important to extract the methylation levels of CpG loci uncovered by the existing methylation array data, especially for the 450 K array data from precious cancer studies.

In these few years, we and other researchers had proposed some DNA methylation prediction models based on SVM, random forest, logistic regression and deep learning [10–17]. A recently proposed random forest model (RF Zhang) predicted methylation rates for bulk ensembles of cells [15], which took comprehensive DNA annotation features into account, including genomic contexts, and tissue-specific regulatory annotations such as DNase1 hypersensitivity sites, histone modification marks, and transcription factor binding sites. DeepCpG was a deep neural network model for predicting the methylation state of CpG dinucleotides in multiple cells based on surrounding sequence components and neighbouring methylation information [13]. EAGLING significantly expanded the 450 K methylation array based on a logistic regression method with neighbouring methylation value and local methylation profiling [10, 18]. Most of the models performed well and could achieve the prediction accuracy close to 90%. However, these works always focused on achieving better overall prediction performance, and could not tell which predicted CpG loci were accurately predicted and which ones were relatively less accurate. Then it is hard for the biologists to select candidates for downstream analysis. Therefore, there is an urgent need to establish precise prediction models that could tell how accurate the predicted methylation level is.

Previous studies have indicated that the methylation level of a CpG locus was correlated with the methylation levels of its neighbouring CpG loci (indicating possible co-methylation), and the methylation marks of the upstream and downstream CpG loci were widely used as important and informative features for prediction [15, 19–21]. Because the distances between the correlated CpG loci are with high degree of variance [22], the neighbouring CpG loci in fixed flanking length could only provide limited information. There is no method that investigates the co-methylated CpG loci without the restriction of flanking length. And this strategy could improve the prediction accuracy for CpG loci that did not have highly correlated neighbouring CpG loci or had no surrounding CpG locus in the defined flanking region.

**Fig. 1:**
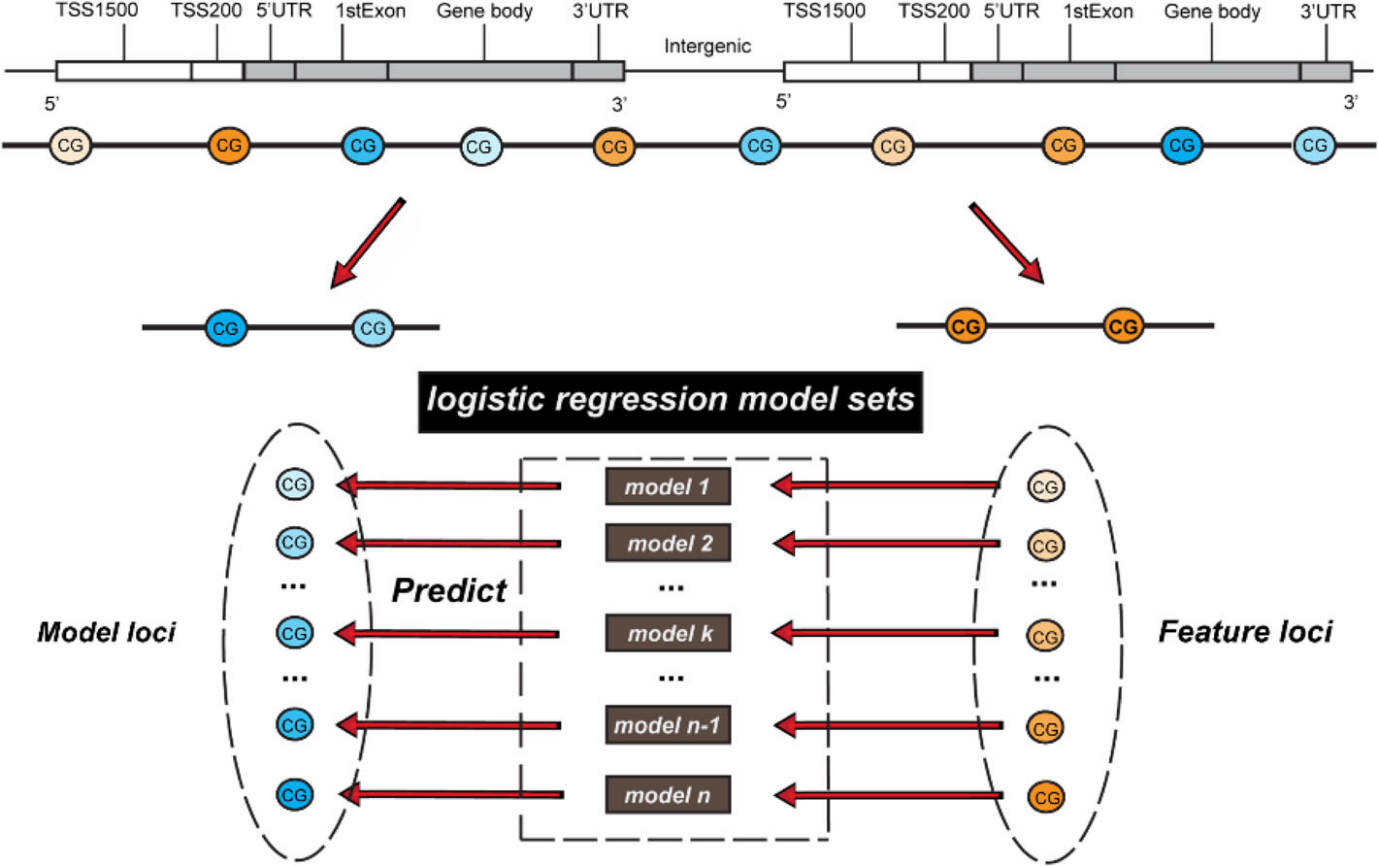
The diagram of our precise prediction models. The CG with yellow color represents the feature locus, the CG with blue color represents the model locus. And the shade of the color indicates the methylation level, the dark color represents hyper-methylation, the light color represents hypo-methylation. The CpG loci covered by EPIC array but not included in 450 K array were defined as the model loci, the loci covered by both EPIC and 450 K array were defined as the feature loci. PretiMeth accurately predicts the methylation levels of model loci by the methylation levels of feature loci

In this study, we proposed to predict the methylation levels of model loci (the target CpG loci to be predicted) by using the methylation levels of feature loci (the CpG loci used for feature selection) and constructed logistic regression model for each model locus (Fig. 1). We called the method as PretiMeth (PREcise predicTIon Models for DNA mETHylation). The EPIC array data were used for prediction model construction. The CpG loci covered by EPIC array but not included in 450 K array were defined as the model loci, the loci covered by both EPIC and 450 K array were defined as the feature loci. For each model locus, the methylation values of its co-methylated CpG locus was finally selected as the prediction feature. The co-methylated locus was defined as a CpG locus whose methylation value was highly correlated with the target CpG locus among different samples. Logistic regression was applied to predict the methylation levels of model loci based solely on their co-methylated loci. In both of the cross-validation and independent data test, PretiMeth demonstrated satisfying performance and outperformed other comparable methods. Besides, our models could provide the prediction accuracy for each specific CpG locus according to the RMSE from cross-validation results. To further utilize and evaluate the prediction results, we divided the model loci into four categories: Super high accurate, High accurate, Medium accurate and Low accurate model loci. For the Super high accurate and High accurate model loci, our proposed models would get very high prediction performance (r = 0.99 and 0.96), and the prediction results were quite consistent with the methylation levels detected with the EPIC array.

Furthermore, we applied our precise prediction models (Super high accurate and High accurate models) on 13 cancers from TCGA and obtained the methylation landscapes for the tumor and normal samples. To identify the markers related to pan-cancer, we analyzed the differentially methylated loci for each cancer, and three CpG loci were found to be hypomethylated in at least 12 cancers. One of the CpG loci, chr16:57798350, was located in the enhancer region, where DNase I and H3K27ac were marked and also bound by a variety of transcription factors (TFs), indicating that it may be a potential therapeutic target for a variety of cancers. In the investigation of the differentially methylated genes, we found 10 genes differentially methylated in at least 10 cancers. The functions of these differentially methylated loci and genes in the development of cancer would be worth for further biological validation.

## Results

### Methylation correlations between model loci and the candidate feature loci

To compare the methylation similarity between the model loci and the three candidate feature loci (including the nearest neighbouring CpG loci, the CpG loci with the most similar flanking sequence, and the co-methylated CpG loci), we calculated the Pearson correlation coefficients between their methylation values based on 665 samples form EPIC array to characterize their methylation correlations.

Previous prediction work using the methylation values from the nearby CpG loci had shown that the nearby loci were closely co-methylated, particularly when the distance was less than 2 kb from each other [15, 19, 20]. Therefore, the correlation results were analysed based on whether the distance was less than 2 kb or not.

Firstly, we restricted to find the nearest neighbouring loci in the 2 kb flanking region of the model loci, and there were 189,582 model loci meeting the requirement. For this part of model loci, the average correlations between the model loci and three candidate feature loci were 0.5299 (the nearest neighbouring loci), 0.6592 (the loci with the most similar flanking sequence), and 0.8658 (the co-methylated loci), respectively (Additional file 2: Figure S1a).

**Fig. 2:**
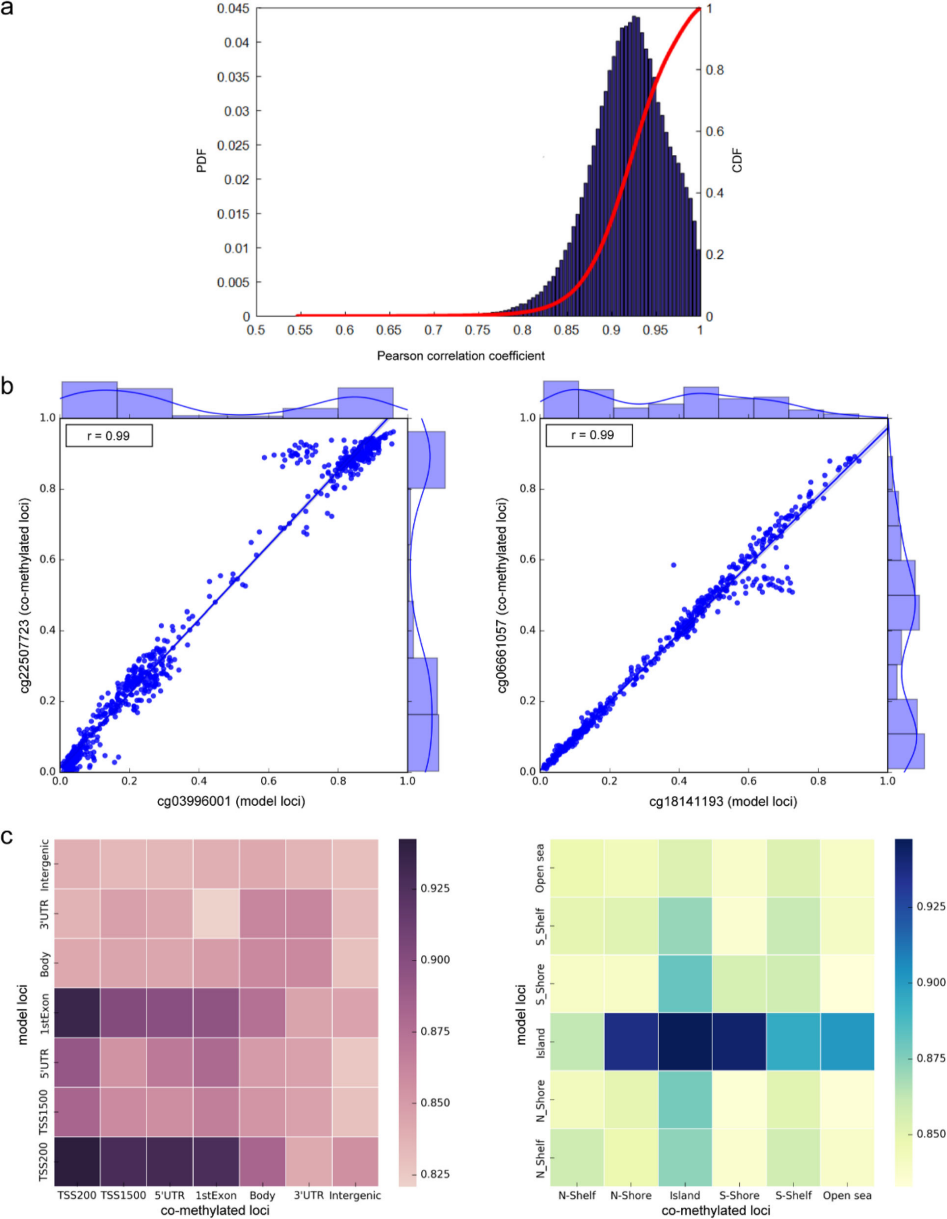
Correlation analysis between different CpG loci. **a** The distribution of correlation coefficients for model loci and their co-methylated loci. The solid red line represents the cumulative distribution function (CDF) and the blue histogram represents the probability density function (PDF) of the Pearson correlation coefficient. **b** The methylation profile between two pairs of model loci and their co-methylated loci. One pair of loci are located in adjacent sequence positions from each other: cg03996001 (chr12:58131766) and cg22507723 (chr12:58131768); Another pair of loci are located at remote sequence positions from each other: cg18141193 (chr16:2610285) and cg06661057 (chr16:2689908). **c** The correlation coefficient matrix between model loci and their co-methylated loci located indifferent genomic regions

Then, we compared the correlation between all 413,719 model loci and the three feature loci without the restrictions of the 2 kb franking range (Additional file 2: Figure S1b; Fig. 2a). We found that the average correlation between the model loci and the nearest neighbouring loci fell to 0.4651. Between the model loci and the loci with the most similar flanking sequence, the correlation was significantly reduced and the average correlation decreased from 0.6592 to 0.5016. In contrast, the correlation with the co-methylated loci remained at a consistently high level (average of 0.8434), and the correlation index between 300,663 model loci and their corresponding co-methylated loci was above 0.8. The significant co-methylation trend was shown between the model loci and their co-methylated loci, which indicated that the methylation values of the co-methylated loci might be more highly informative for prediction than the other two types of feature loci.

Besides, we investigated the location distances between all the model loci and their co-methylated loci, and found that ~ 95% of distances were larger than 2 kb, which indicated that the co-methylated loci not only exist in the nearby regions of model loci but also could exist in two distal regions (Fig. 2b). We further evaluated the profile of the correlation between model loci and their co-methylated loci based on the different regions of the genome (Fig. 2c). The model loci and their co-methylated loci both located in the gene promoter region showed a higher correlation than other regions (especially in TSS200 and 1stExon). When investigating the correlation related to CGI regions, the pair loci both located in the CpG island region showed a higher correlation.

### Model construction

**Fig. 3:**
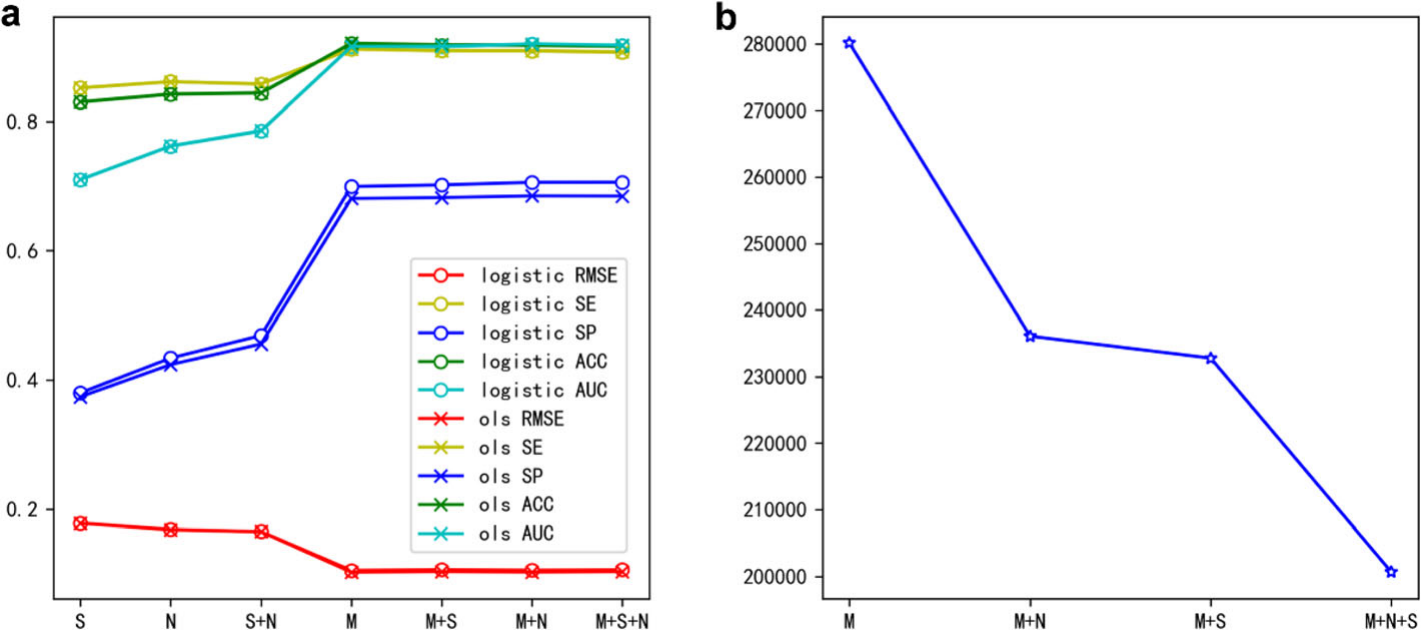
The impact of different features and algorithms for constructing prediction models. **a** Comparison of predicted performance based on 5-fold cross-validation using different feature combinations and algorithms. **b** The number of predictable model loci using different combination of candidate features. (M: the co-methylated loci; N: the nearest neighbouring loci; S: the loci with the most similar flanking sequence)

To establish separate prediction models for each CpG locus, we used the methylation values of the three candidate feature loci as the prediction features. Due to the high computational cost of algorithms like random forest and deep learning etc., only the Ordinary Least Squares (OLS) and Logistic Regression (LR) algorithms were considered to construct models. The 5-fold cross-validation results on training data were compared based on the different feature combinations and regression models (Fig. 3a).

For the model choice, the prediction results were quite comparable based on Fig. 3a. The performance of LR model was slightly better than the OLS model, and the output value of the logistic regression method was more in line with the definition of methylation level. Therefore, the logistic regression model was finally selected for model construction.

For feature selection, we found that the contribution of the co-methylated loci was significantly higher than the other two types of features, which was consistent with the conclusions of our correlation analysis. Although the performance of the model could increase slightly when the three features were all applied. However, only the fewer model loci could be predicted when more features were used, due to the missing values existed in the 450 K array data. In each regression model, as long as one independent variable (the methylation level of a feature locus), the dependent variable (the methylation level of a model locus) could not be calculated. Taking one 450 K array data used for prediction as an example, the number of predictable model loci was about 280,000 when only one feature was applied for prediction, and the number of predictable model loci was reduced by more than 40,000 when two features were applied, while the number was reduced to 200,000 when three features were applied (Fig. 3b).

Therefore, considering the balance between the accuracy and practicality of the prediction method, we only used the feature of the co-methylated loci to develop PretiMeth based on logistic regression algorithm.

### Performance evaluation

After the model construction of PretiMeth, the performance was evaluated in cross-validation, independent testing and cross-platform evaluation.

1. **Cross-validation performance and model categorization**

**Fig. 4:**
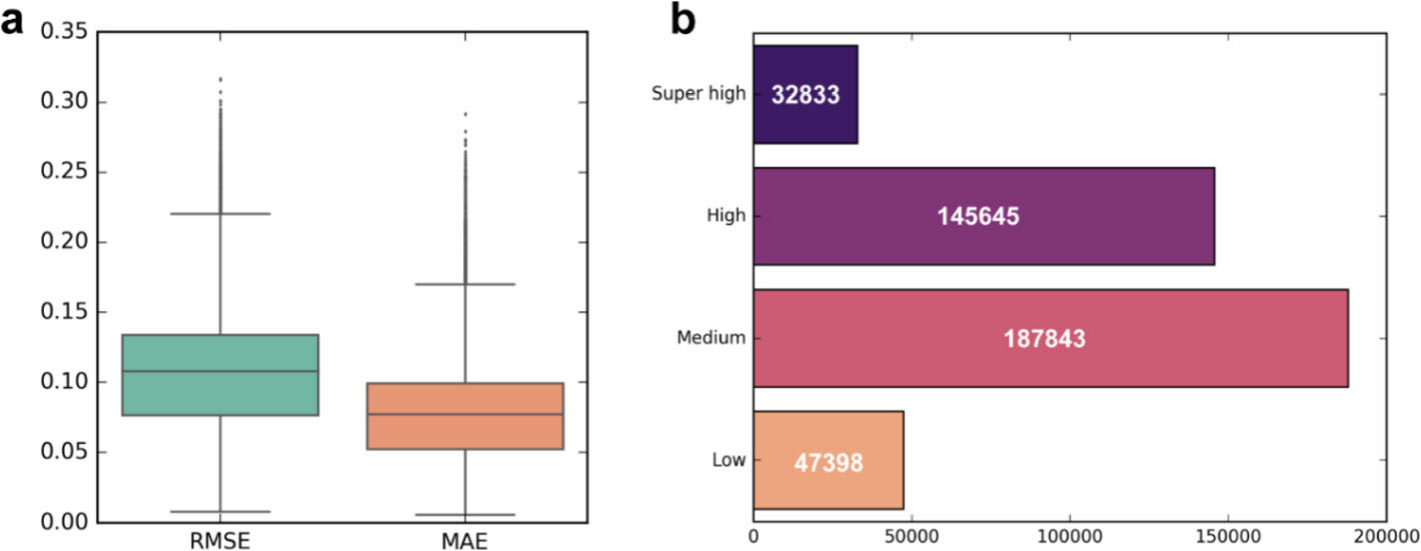
The prediction performance in 5-fold cross-validation and the number of loci for four categories with different accuracies. **a** The performance of RMSE and MAE on the 5-fold cross-validation of all single-locus models. **b** The number of four categories of models based on RMSE partitioning

The cross-validation performance of each single CpG locus model was evaluated on 665 EPIC samples. For each model, the model performance was evaluated by 5-fold cross-validation on 665 samples. The evaluation metrics include root mean square error (RMSE) and mean absolute error (MAE). We calculated RMSE and MAE in 5-fold cross-validation for all models (Fig. 4a). The average values of them were 0.1054 and 0.0766, respectively. The model shows good prediction performance in cross-validation, which proves that our single-locus modeling strategy is effective (Additional file 2: Figure S2).

The advantage of our single-locus modeling compared with the previous general models is that our precision model could tell how accurate the predicted methylation levels of the CpG loci were, which means one can know which CpG locus is accurately predicted and which is relatively unreliable. Therefore, we used the RMSE of the cross-validation results to assess the accuracy of the predictions for each single-locus model. But there are actually no fixed restrictions on the division of the models, the division of the models mainly lies in the user’s personalized judgment on the accuracy of the model and the task requirements. And we recorded eight performance indicators (Pearson correlation coefficient, RMSE, MAE, SP, SE, MCC, ACC, and AUC) from cross-validation for users’ reference. Here, based on RMSE, the models were divided into four categories as Super high accurate model (RMSE < 0.05), High accurate model (0.05 ≤ RMSE < 0.1), Medium accurate model (0.1 ≤ RMSE < 0.15) and Low accurate model (RMSE ≥ 0.15). Each category contains 32,833, 145,645, 187,843 and 47,398 CpG loci, respectively (Fig. 4b).

2) **Prediction performance on independent data**

To further verify the performance of our proposed precision models, we applied these models on other 139 independent test samples and got the average values of the performance indicators. For all the four categories of models, the overall average performances were with small RMSE (0.0989 ± 0.0375) and MAE (0.0694 ± 0.0244), while high Pearson correlation coefficient (0.9309 ± 0.0375), SP (0.8711 ± 0.0697), SE (0.9489 ± 0.0445), MCC (0.8263 ± 0.1002), ACC (0.9283 ± 0.0478) and AUC (0.9697 ± 0.0320), which demonstrated the satisfying performance of our models (Table 1).

**Table 1:** The performance of methylation prediction based on different categories of prediction models

Model	R	RMSE	MAE	SE	SP	MCC	ACC	AUC
All	0.93	0.10	0.07	0.95	0.87	0.83	0.93	0.97
Super high accurate	0.99	0.03	0.02	0.99	0.99	0.99	0.99	0.99
High accurate	0.96	0.07	0.05	0.98	0.93	0.92	0.97	0.98
Medium accurate	0.88	0.11	0.08	0.93	0.82	0.76	0.90	0.95
Low accurate	0.79	0.15	0.11	0.86	0.80	0.66	0.85	0.90

Moreover, three other independent test sets (two tumor samples and one normal sample) from the NCBI GEO database, were used to compare PretiMeth with EAGLING [10, 18] and Impute knn [23]. The comparison results were listed in Additional file 2: Table S1. PretiMeth demonstrated more accurate prediction results on the three independent test sets than the two models.

**Fig. 5:**
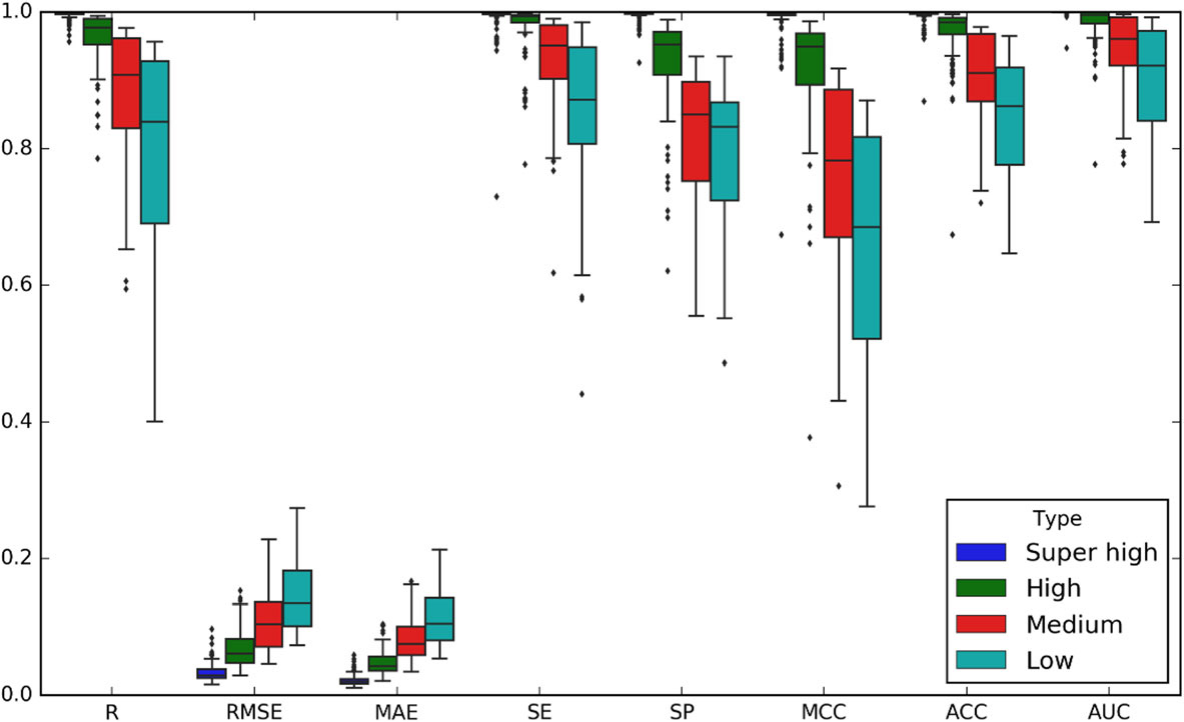
The performance of four categories of models in independent test sets, including Super high, High, Medium and Low accurate models

Then we observed the performance of the four categories of models on the 139 independent testing sets. The box-plot results were shown in Fig. 5. One could see that the Super high accurate and High accurate models achieved extremely high prediction accuracy, while the Medium accurate models also achieved high accuracy (ACC ≥ 0.9) to the other general prediction models in the state of art. It is worth noting that for the Super high accurate model, the correlation coefficient between the predicted methylation value and the methylation values derived with EPIC array reached 0.99 ( RMSE = 0.03).

3) **Prediction performance across platforms**

**Fig. 6:**
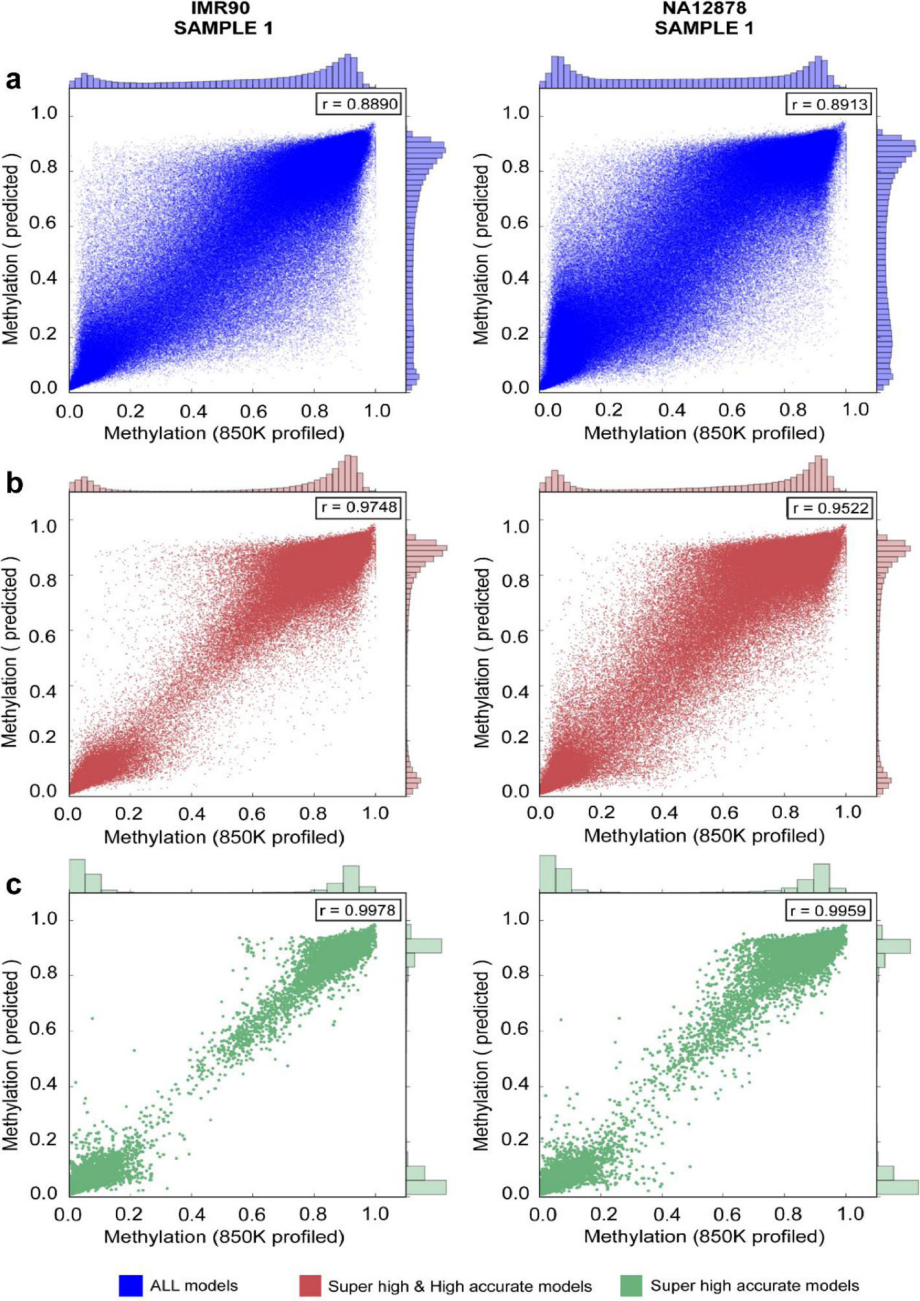
Comparison of the predicted methylation levels and the methylation levels profiled by EPIC technology in IMR90 sample 1 and NA12878 sample 1 from (**a**) all prediction models, (**b)** Super high and High accurate models, and (**c**) only Super high accurate models

The Super high accurate and High accurate models of PretiMeth maintained high prediction performance across platforms. To verify the expansion ability of the model from 450 K array to EPIC array, we applied our models to predict the methylation levels of the model loci (measured by EPIC array) by the methylation levels of the co-methylated loci (measured by 450 K array). The average prediction results were shown in Table 2, and the scatter plot comparing the predicted methylation values with the methylation values detected by EPIC data were shown in Fig. 6 (Additional file 2: Figure S3). The previous studies have evaluated the potential of a joint analysis of 450 K data and EPIC data [24, 25]. And the stable high prediction performance of the Super high accurate and High accurate model indicates that they can be applied to expand the existing 450 K data and support the joint analysis.

**Table 2:** The prediction performance in the cross-chip evaluation

Cell line	Model	R	RMSE	MAE	SE	SP	MCC	ACC	AUC
IMR90	Super high accurate	0.99	0.03	0.02	0.99	0.99	0.99	0.99	0.99
	High accurate	0.96	0.08	0.05	0.99	0.91	0.92	0.98	0.99
	Medium accurate	0.80	0.16	0.12	0.88	0.75	0.64	0.83	0.90
	Low accurate	0.61	0.21	0.16	0.70	0.74	0.45	0.72	0.79
NA12878	Super high accurate	0.99	0.04	0.02	0.99	0.99	0.99	0.99	0.99
	High accurate	0.93	0.11	0.08	0.98	0.81	0.82	0.93	0.97
	Medium accurate	0.82	0.17	0.13	0.88	0.80	0.67	0.83	0.92
	Low accurate	0.71	0.20	0.16	0.74	0.84	0.56	0.81	0.87

Furthermore, we applied our Super high accurate and High accurate models to WGBS data, using the methylation values of the co-methylated loci to predict the methylation levels of the model loci (the methylation values were both measured by WGBS). We found that the Super high accurate and High accurate models achieved accuracies of 95% and 94% on different samples (Additional file 2: Table S2), which also indicated the potential of applying our model to the expansion from 450 K array to the WGBS-scale data.

### Application to the TCGA data

Here, we retrieved Illumina 450 K array data for 13 cancers in the TCGA database, including 667 normal samples and 5275 tumor samples. For each 450 K data, we applied our PretiMeth model to predict the methylation levels of the model loci. On average, we obtained about 297,738 model loci based on the 450 K data for each cancer, and 132,391 of them belonged to the Super high accurate and High accurate models (Additional file 2: Table S3). We performed differential methylation analysis and summarized significant DMLs (mean difference of DNA methylation > 0.1 and q < 0.05) on the prediction results. To take advantage of PretiMeth (indicating the prediction accuracy of each single CpG locus), only the CpG loci predicted based on the Super high accurate and High accurate models were applied for DML analysis to ensure the reliability of the analysis.

**Fig. 7:**
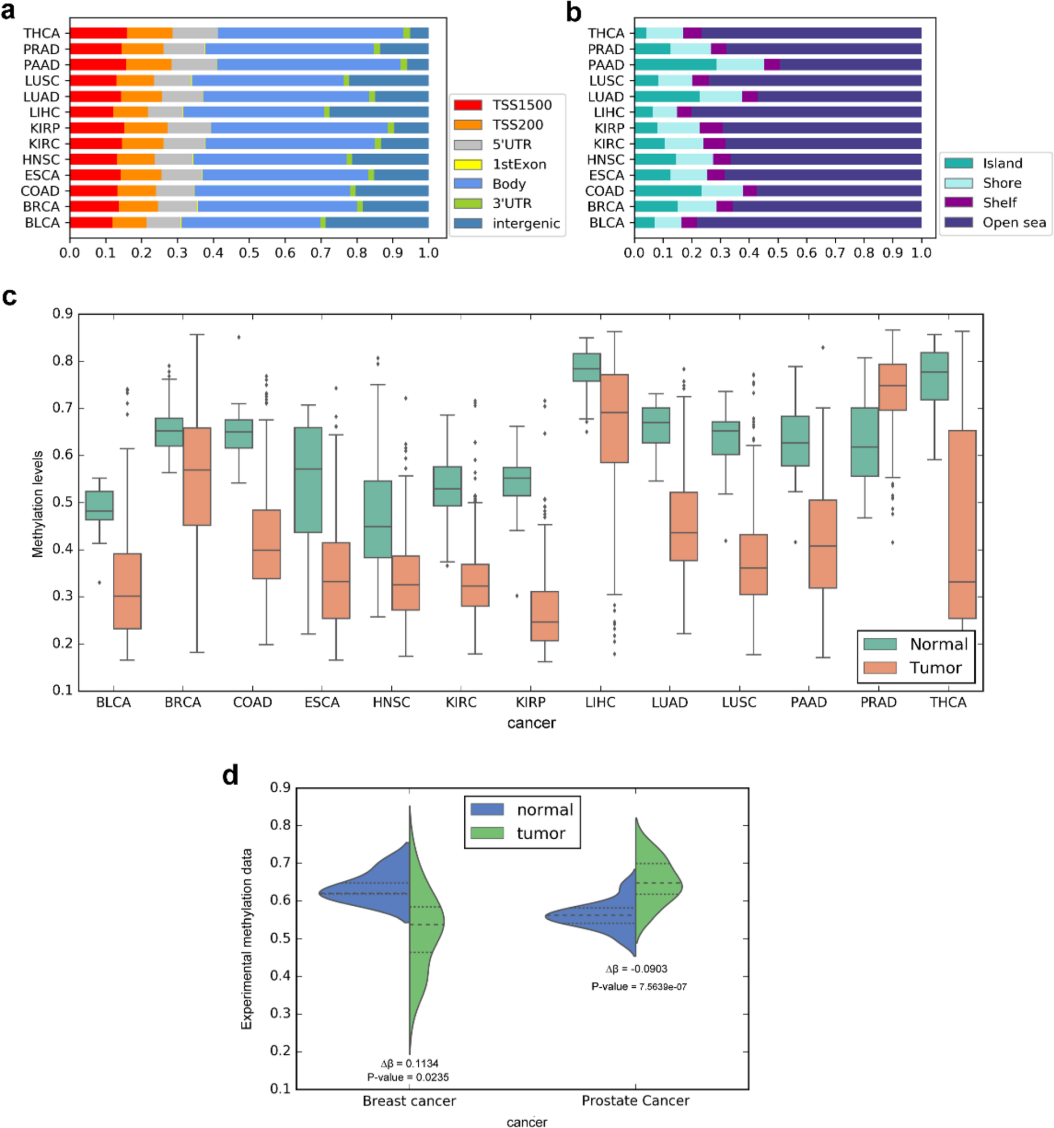
The differential methylation landscapes for the 13 cancers. **a** The proportions of DMLs in different genomic regions. **b** The proportions of DMLs in CGI associated regions. **c** Probe chr16:57798350, located in the body region of the KIFC3 gene, showed significant hypomethylation (the methylation level of the locus in tumor samples were lower than those in normal samples) in 12 cancers based on predicted methylation data. **d** Probe chr16:57798350 showed the same differential methylation trend in two independent EPIC datasets of prostate cancer and breast cancer

In our results, most of these DMLs were located in non-promoter regions (gene body, 3′UTR, and intergenic region; Fig. 7a) and non-CGI regions (shore, shelf, and open sea; Fig. 7b) in each cancer. This also reflected the coverage of the EPIC array design, which provided more methylation information of loci in the remote regulatory region [9, 26]. Intriguingly, we found that three methylation probes were identified as DMLs in all 13 cancers, including chr3:167293827, chr5:2276656, and chr16:57798350. Among them, chr3:167293827 was located in the intergenic region and chr5:2276656 was located in the body region of WDR49, which were both hypomethylated in all 13 cancers (Additional file 2: Figure S4). The probe chr16:57798350 located in the body region of KIFC3 was significantly hypomethylated in 12 cancers except for prostate adenocarcinoma (Fig. 7c).

Previous work had shown that KIFC3 could play important roles in HCC invasion and metastasis [27], and increased KIFC3 expression levels had been associated with docetaxel- and paclitaxel-resistant breast cancer cells [28]. Therefore, we curiously observed the region including chr16:57798350 in the UCSC genome browser [29] and found that the region is marked by DNase I and an active enhancer marker H3K27ac (Additional file 2: Figure S5a). Also, we checked the chromatin status region of the roadmap in the WashU Epigenome Browser [30] (Additional file 2: Figure S5b), and found that they were annotated as Genic enhancers, Enhancers and Strong transcription in different normal cells or tissues. And more than 10 TFs were bound to this enhancer region (Additional file 2: Figure S5a). Some of the TFs have been reported to play key roles in the process of cancers [31–36]. As the methylation changes of an enhancer region can be owing to gain or loss of some transcription factor bindings (TFs) [37–39], we suspected that this enhancer region may be a potential therapeutic target for a variety of cancers.

Moreover, we downloaded two independent datasets of prostate cancer and breast cancer from the GEO database to evaluate whether the locus chr16:57798350 was also differentially methylated. In the differential methylation analysis based on the experimental data, the locus chr16:57798350 showed hypomethylation in breast cancer (∆β = 0.1134, P = 0.0235) and hypermethylation in prostate cancer (∆β =  − 0.0903, P = 7.5639*e*^−07^), which were consistent with our above results based on precise prediction data (Fig. 7d).

To explore the DMGs for each cancer, there were 10 genes simultaneously differentially methylated in at least 10 cancers, and we defined them as pan-cancer differentially methylated genes (Additional file 2: Table S4). Six of these genes have been reported to be associated with cancers, including LOC284933 [40], BOD1L2 [41], MIR7515 [42–44], ZNF729 and ZNF479 [45–47], and MKL1 [48–52]. Among them, the LOC284933 gene was found to be differentially methylated in 12 cancers. LOC284933 is an RNA Gene and affiliated with the ncRNA class. Until now, there were few reports on the role of LOC284933 in cancers and only one study reported the loss of 22q13.31–13.32 region including LOC284933 was significantly associated with the presence of ovarian family history [40]. The other four genes, i.e. LINC01246, MIR7515HG, LOC100506384, and MKL1, there was still no obvious evidence about their relationship with any cancers. Therefore, further study about the abnormal methylation of these genes are required to decipher their potential roles in the development of cancers.

## Discussion

Here, a method named PretiMeth was proposed to estimate the DNA methylation levels at single CpG resolution. PretiMeth was based on a logistic regression algorithm to achieve single locus modeling. Importantly, PretiMeth picked up on potential co-methylated loci by calculating the methylation correlation between distant CpGs to improve the prediction performance. The performances of cross-validation and independent testing on the EPIC array data indicated that PretiMeth could achieve high accuracy. In the cross-platform performance evaluation, the Super high accurate and High accurate models performed quite well on both array and WGBS data. Furthermore, we applied our PretiMeth to The Cancer Genome Atlas data and expanded the existing 450 K array data of 13 cancers. The intensive results of differential methylation analysis on pan-cancer demonstrated that our method could offer reliable expanded methylation information for downstream analysis in a cost-effective manner. Overall, our results validated that PretiMeth not only achieved performance comparable to other previous models (ACC > 90%) based on only one significant feature, but also had the capability to indicate the estimation accuracy for each CpG locus.

The previous studies on methylation prediction have shown that the inclusion of CGIs, genomic location, DNase I hypersensitization sites, and histone modifications can produce accurate prediction results [15, 53–56]. However, in practical applications, obtaining the corresponding necessary information is usually not feasible. In our algorithm, it could slightly improve the prediction performance by adding the methylation marks of neighbouring flanking CpG loci and the CpG loci with the most similar flanking sequence component. However, the more features were applied, the less CpG loci could be predicted. Therefore, PretiMeth was constructed based on only one prediction feature about the co-methylated locus. This not only simplified the model construction but also improved prediction for CpG loci that did not have highly correlated neighbouring CpG loci or had no surrounding CpG locus in the defined flanking region.

The prediction accuracy information for each CpG locus provides the chance to select more reliable results for relevant bioanalysis. When applying our model to TCGA data, we only focused on the CpG loci derived from the Super high accurate and High accurate models to implement the differential methylation analysis between the tumor and normal samples. The pan-cancer analysis based on the most precise prediction methylation data showed that the locus Chr6:57798350 was differentially methylated in 12 cancers. This highlight locus was located in the enhancer region, which was marked with DNase I and H3K27ac. This region was also bound by many TFs that have been reported to be associated with a variety of cancers. Moreover, the abnormal methylation of this locus was also confirmed in other two experimental data, which confirmed the reliability of precise prediction results and further suggested that it might be a potential therapeutic target for cancers.

Based on our PretiMeth model, we could accurately predict the methylation level of some EPIC-covered loci by using the methylation level of 450 K-covered loci. Therefore, it would be quite meaningful to investigate which CpG loci could be accurately predicted based on the 450 K array or EPIC array, and then there is no need to add them on the array chip. Therefore, our PretiMeth can be probably used for reference in the probe set design when the DNA methylation beadchip updates.

An important question of interest is whether PretiMeth could be applied to the whole-genome expansion. In this study, we developed our precise models on EPIC array data. And achieved good prediction performance for the EPIC newly covered CpG loci based on the CpG loci covered by both 450 K and EPIC. In theory, the strategy could generalize to whole-genome prediction. For example, we can divide the whole-genome CpG loci into two parts: the CpG loci covered by EPIC array are represented as feature loci, and the remaining CpG loci are represented as model loci. Before the implementation, the consistency between the arrays and the WGBS data and the limited available datasets should be analyzed systematically first, and it would be our work in the next step.

## Conclusions

In this study, we reported PretiMeth, a method for constructing precise prediction models for each single CpG locus, based on only one significant methylation mark. PretiMeth used a single-locus modeling strategy and could provide the evaluation of the prediction accuracy for each single CpG locus, which would facilitate the candidate selection for the following biological applications. Meanwhile, our findings supported the idea that the methylation value of the co-methylated locus is very important for the methylation prediction work.

## Methods

### Sample collection

To establish the precise prediction models at CpG site resolution, we collected the available EPIC array data from the NCBI GEO database. Among them, the methylation landscapes of 804 samples measured by EPIC (406 from tumors and 398 from non-tumors) were used for model construction (665 samples for cross-validation and the remaining 139 samples for independent testing). Moreover, there were 3 additional samples measured by EPIC that were used to the comparison of the prediction performance between PretiMeth and the other two methods. For evaluating the cross-platform prediction performance, there were 7 samples (measured by both 450 K and EPIC arrays) for cross-chip evaluation and another 2 samples for evaluating the prediction performance on WGBS data. Overall, 816 samples were used during the model construction and the testing of model performance, covering more than 30 cell lines or tissue types (Additional file 1).

For the model application, the 450 K array data of the TCGA database were downloaded. The cancers with at least 10 normal samples were selected in our study. Finally, 13 cancers with a total of 667 normal samples and 5275 cancer samples remained (Additional file 2: Table S5). And another two independent datasets of prostate cancer and breast cancer were used to validate the highlight DML results (Additional file 1).

Both EPIC and 450 K array data were quantile normalized before the following analysis.

### Prediction model

#### CpG locus division

For building single-locus prediction models, the EPIC array data were applied, the CpG loci covered by EPIC but not included in 450 K array were defined as model loci, the loci covered by both EPIC and 450 K array were defined as feature loci. Totally, there were 413,719 model loci and 450,137 feature loci.

#### Features for prediction

Three kinds of candidate features were used for model construction: the methylation value of the nearest neighbouring CpG locus, the methylation value of the co-methylated CpG locus, and the methylation value of the CpG locus with the most similar flanking sequence. The definitions of three kinds of feature loci were:

- the nearest neighbouring CpG locus: the feature loci located closest to the model loci on the same chromosome.
- the co-methylated CpG locus: the feature loci sharing the most similar methylation pattern with model loci in the EPIC samples.
- the CpG locus with the most similar flanking sequence: the feature loci sharing the most similar flanking sequence-component pattern with model loci.

To characterize the co-methylation pattern between CpG loci across samples, we constructed a multidimensional vector for each locus to measure the co-methylation pattern between CpGs across samples: ${\beta}_{CpGi}=\left\{{\beta}_i^1,{\beta}_i^2,\dots, {\beta}_i^n\right\}$, *β*_*CpGi*_ denotes the vector of the methylation value of the i-th locus in all n samples and ${\beta}_i^k,k=1,2,\dots, n$ represents the methylation value of the i-th locus in the k-th samples.

To characterize the sequence composition pattern across CpGs, we extracted 340 sequence features in the range of 200 bp flanking range of the i-th locus, including all 1- to 4-mers occurrence frequencies: ${Seq}_{CpGi}=\left\{\ {OC}_{CpGi}^{1- mers},{OC}_{CpGi}^{2- mers},{OC}_{CpGi}^{3- mers},{OC}_{CpGi}^{4- mers}\right\}$. For example, the 1-mers occurrence frequencies: ${OC}_{CpGi}^{1- mers}=\Big\{ num(A)/{ASC}^{1- mers}, num(T)/{ASC}^{1- mers}, num(C)/{ASC}^{1- mers}, num(G)/{ASC}^{1- mers}$ }, *ASC*^1 − *mers*^ = *num*(*A*) + *num*(*T*) + *num*(*C*) + *num*(*G*), *num*(*N*) represents the number of occurrence of base N, N represents any nucleotide, i.e. A/G/T/C.

The methylation similarity *PearsonMeth*_*ij*_ and the sequence similarity *PearsonSeq*_*ij*_ were measured by the Pearson correlation coefficient between model loci and feature loci:

$$ {PearsonMeth}_{ij}=\frac{\mathit{\operatorname{cov}}\left({\beta}_{CpGi},{\beta}_{CpGj}\right)}{\sigma_{\beta_{CpGi}}\cdotp {\sigma}_{\beta_{CpGj}}},{\beta}_{CpGi}\ne {\beta}_{CpGj} $$

$$ {PearsonSeq}_{ij}=\frac{\mathit{\operatorname{cov}}\left({ Se q}_{CpGi},{ Se q}_{CpGj}\right)}{\sigma_{Se{q}_{CpGi}}\cdotp {\sigma}_{Se{q}_{CpGj}}},{ Se q}_{CpGi}\ne { Se q}_{CpGj} $$

where *cov* represents the covariance operation and *σ* represents the standard deviation operation.

After calculating the correlation coefficient between the model loci and the feature loci, we select two specific feature loci for each model loci, which shared the highest methylation pattern correlation coefficient and sequence composition pattern correlation coefficient respectively:

$$ \left({CpG}_i,{CpG}_p\right):{PearsonMeth}_{ip}=\underset{j=1,2,\dots, m}{\max }{PearsonMeth}_{ij} $$

$$ \left({CpG}_i,{CpG}_q\right):{PearsonSeq}_{iq}=\underset{j=1,2,\dots, m}{\max }{PearsonSeq}_{ij} $$

where *CpG*_*i*_ represents the i-th model loci, *CpG*_*p*_ represents the co-methylated loci p for *CpG*_*i*_, and *CpG*_*q*_ represents the loci q with the most similar flanking sequence for *CpG*_*i*_.

Finally, the co-methylated CpG locus, the CpG locus with the most similar flanking sequence, and the nearest neighbouring CpG locus will be used as three methylation marks for analysing in this study. The three types of features are defined only based on the training samples.

Besides, we observed the similarities between the model loci and feature loci distributed in different functional regions. Based on the UCSC annotation, the loci were classified into TSS200, TSS1500, 5’UTR, 1st Exon, Body and 3’UTR. Related to this last classification, categories included TSS200 represents the region between 0 and 200 bases upstream from the transcriptional start site (TSS); TSS1500 represents the region between 200 and 1500 bases upstream from the transcriptional start site (TSS); 5’UTR included the region between the TSS and the start site (ATG); CpGs within the first exon of a gene were considered as 1st Exon category; CpGs downstream the first exon including intronic regions until the stop codon, were classified as gene body; CpGs located downstream the stop codon until the poly A signal were considered as 3’UTR; and CpGs that were not classified in any of the previous categories were annotated as intergenic. When multiple genes or TSS were associated with a CpG locus, category prioritization was applied following a 5′-prime to 3′-prime criteria (TSS200 > TSS1500 > 5’UTR > 1st Exon > Body > 3’UTR > Intergenic). Additional criteria included the location of the CpG loci relative to the CpG island (open sea, island, shore, shelf) [9].

#### Regression prediction model

The logistic regression algorithm and the ordinary least squares algorithm were respectively developed to predict the methylation levels of model loci using the methylation levels of feature loci in training data. Six hundred sixty-five samples from the 804 samples (measured by EPIC array) were used to implement a 5-fold cross-validation strategy to construct the prediction model, and the remaining 139 samples were used as independent testing set for model performance evaluation.

Let variable *β*_*CpGl*_ represents the methylation level of the nearest neighbouring loci l, *β*_*CpGi*_ represents the methylation level of the i-th model loci, *β*_*CpGm*_ represents the methylation level of the matched co-methylated loci m, and *β*_*CpGn*_ represents the methylation level of the matched loci n with most similar sequence.

For the i-th model loci, we first constructed an independent logistic regression model to predict its specific methylation levels in samples:

$$ {\beta}_{CpGi}=\mathrm{F}\left(\mathrm{X}\right)=P\left(X\le x\right)=\frac{1}{1+{e}^{-\left(x-u\right)/y}} $$

Where *u* represents the position parameter, *y* > 0 represents shape parameter and *x* = { *β*_*CpGl*_, *β*_*CpGm*_, *β*_*CpGn*_}.

The methylation level predicted by the logistic regression model for the i-th model locus in the k-th sample is:

$$ {\beta}_i^k(LR)=\frac{\mathit{\exp}\left({w}_i\cdotp {x}^k+{b}_i\right)}{1+\mathit{\exp}\left({w}_{\mathrm{i}}\cdotp {x}^k+{b}_i\right)}=\mathrm{sigmoid}\left({w}_i\cdotp {x}^k+{b}_i\right) $$

Where ${x}^k=\left\{\ {\beta}_{CpGl}^k,\right.\left.{\beta}_{CpGm}^k,{\beta}_{CpGn}^k\right\}$, ${\beta}_{CpG\ast}^k$ represents the experimental methylation levels of the matched particular loci in the k-th sample for *CpGi*, (*w*_*i*_, *b*_*i*_) is the fitting parameter of the logistic regression model for *CpGi*, and the region of ${\beta}_i^k(LR)$ represents a number in [0,1] which defines the probability of that *CpGi* being methylated.

Then we constructed an independent ordinary least squares model to compare with the logistic regression model. The methylation level predicted by the ordinary least squares model for the i-th model locus in the k-th sample is:

$$ {\beta}_i^k(OLS)={\alpha}_i\cdotp {x}^k+{c}_i $$

Where (*α*_*i*_, *c*_*i*_) is the fitting parameter of the ordinary least squares model for *CpGi* and we limited the region of ${\beta}_i^k(OLS)$ to a number in [0,1] which defines the probability of that *CpGi* being methylated.

#### The evaluation of prediction performance

To evaluate and compare the predictive performance of models, we used evaluation metrics include Pearson correlation coefficient (R), Root-Mean-Square-Error (RMSE), Mean-Absolute-Error (MAE), Sensitivity (SP), Specificity (SE), Matthew’s correlation coefficient (MCC), Accuracy (ACC) and AUC (Area Under ROC Curve). The calculation formulas for these indicators are as follows:

$$ \mathrm{RMSE}\left(Y,{Y}^0\right)=\sqrt{\frac{\sum_{i=1}^n{\left(Y-{Y}^0\right)}^2}{n}} $$

$$ \mathrm{MAE}\left(Y,{Y}^0\right)=\frac{1}{n}\sum \limits_{i=1}^n\left|Y-{Y}^0\right| $$

where Y represents the predicted value of the methylation level and *Y*^0^ represents the detected value with array or WGBS technique.

For calculating SP, SE, MCC, and ACC, we defined the methylation status as + 1 if the methylation value is larger than 0.5, and the methylation status as − 1 otherwise.

$$ \mathrm{SE}=\frac{TP}{TP+ FN}\ \mathrm{SP}=\frac{TN}{TN+ FP} $$

$$ \mathrm{ACC}=\frac{TP+ TN}{TP+ FN+ TN+ FP} $$

$$ \mathrm{MCC}=\frac{TP\ast TN- FP\ast FN}{\sqrt{\left( TN+ FN\right)\ast \left( TN+ FP\right)\ast \left( TP+ FN\right)\ast \left( TP+ FP\right)}} $$

Here, TN, TP, FN and FP represented the number of true-negatives, true-positives, false-negatives and false-positives, respectively.

### The identification of DML and DMG

A Welch’s t-test was used to find differentially methylated loci (DMLs) between the tumor and normal samples. For normal and cancer samples in each cancer, the limma package in R was used to reduce the batch effects for normal samples and cancer samples respectively. The *P*-values were subjected to Benjamini-Hochberg correction for multiple hypothesis testing to calculate q-value.

To define a DML, two conditions were required:1) q values were less than 0.05; 2) the differences in average β values were larger than 0.1. Genes whose promoter regions included DML were defined as differentially methylated genes (DMGs). A promoter region of a gene is defined as a collection of TSS200 (0–200 bps upstream the TSS), TSS1500 (200–1500 bps upstream the TSS), 5’UTR, and 1st Exon regions.

## Supplementary information

**Additional file 1:** A detailed note of the samples used in this study.

**Additional file 2: Table S1.** The comparison of prediction performance based on three independent test sets. **Table S2.** The prediction performance in WGBS data. **Table S3.** The number of all analyzed methylation loci and DMLs in each cancer. **Table S4.** The differentially methylated genes related to pan-cancer. **Table S5.** The number of normal and tumor samples for each cancer. **Figure S1.** The distribution of correlation coefficients for model loci and the candidate feature loci. **Figure S2.** The classification performance of 413,719 single-locus models on cross-validation. **Figure S3.** Scatter plotting the predicted methylation levels and the methylation levels profiled by 850 K technology in other samples of IMR90 and NA12878. **Figure S4.** The probe chr3:167293827 and chr5:2276656 showed significant hypomethylation (the methylation level of the locus in tumor samples were lower than those in normal samples) among all 13 cancers based on predicted methylation data. **Figure S5.** Annotations of the enhancer region we found.

## Data Availability

All data analysed during the current study are available in the TCGA (https://cancergenome.nih.gov/) and NCBI’s GEO (https://www.ncbi.nlm.nih.gov/geo/). The accession numbers of data are listed in Additional file 1. The Source codes of PretiMeth and model parameter files have been provided as an open source available at https://github.com/JxTang-bioinformatics/PretiMeth.

## Abbreviations

450 K: Illumina Infinium Methylation450K BeadChip
EPIC(850 K): Illumina Infinium MethylationEPIC BeadChip
GWAS: Genome wide association study
WGBS: Whole genome bisulfite sequencing
TSS: Transcription start site
TFs: Transcription factors
RMSE: Root-mean-squared error
DML: The differentially methylated loci
DMG: The differentially methylated genes
GEO: Gene expression omnibus
NCBI: National Center for Biotechnology Information
TCGA: The Cancer Genome Atlas data
