# Supplementary material for: PretiMeth: precise prediction models for DNA methylation based on single methylation mark
Source: BMC Genomics. 2020 May 15;21:364. doi: 10.1186/s12864-020-6768-9 (PMC7227319; doi:10.1186/s12864-020-6768-9)
Supplement: Supplementary file 2 — Additional file 2: Table S1. The comparison of prediction performance based on three independent test sets. Table S2. The prediction performance in WGBS data. Table S3. The number of all analyzed methylation loci and DMLs in each cancer. Table S4. The differentially methylated genes related to pan-cancer. Table S5. The number of normal and tumor samples for each cancer. Figure S1. The distribution of correlation coefficients for model loci and the candidate feature loci. Figure S2. The classification performance of 413,719 single-locus models on cross-validation. Figure S3. Scatter plotting the predicted methylation levels and the methylation levels profiled by 850 K technology in other samples of IMR90 and NA12878. Figure S4. The probe chr3:167293827 and chr5:2276656 showed significant hypomethylation (the methylation level of the locus in tumor samples were lower than those in normal samples) among all 13 cancers based on predicted methylation data. Figure S5. Annotations of the enhancer region we found. [file 12864_2020_6768_MOESM2_ESM.docx]

**PretiMeth：precise prediction models for DNA methylation based on single methylation mark**

Jianxiong Tang^1^, Jianxiao Zou^1^, Xiaoran Zhang^1,3^, Mei Fan^4^, Qi Tian^1^, Shuyao Fu^1^, Shihong Gao^1^, Shicai Fan^1,2^*

^1^School of Automation Engineering, University of Electronic Science and Technology of China, Chengdu,611731, China

^2^Center for Informational Biology, University of Electronic Science and Technology of China, Chengdu, 611731, China

^3^Department of Automation, Tsinghua University, Beijing, 100084, China

^4^Chengdu Women's and Children's Central Hospital, School of Medicine, University of Electronic Science and Technology of China, Chengdu 611731, China

*Correspondence: shicaifan@uestc.edu.cn

**Contents**

**Table S1. The comparison of prediction performance based on three independent test sets.**

**Table S2. The prediction performance on WGBS data.**

**Table S3. The number of all analyzed methylation loci and DMLs in each cancer.**

**Table S4. The differentially methylated genes related to pan cancers.**

**Table S5. The number of normal and tumor samples for each cancer.**

**Figure S1. The distribution of correlation coefficients for model loci and the candidate feature loci.**

**Figure S2. The classification performance of 413,719 single-locus models on cross-validation.**

**Figure S3. Scatter plotting the predicted methylation levels and the methylation levels profiled by 850K technology in other samples of IMR90 and NA12878.**

**Figure S4. The probe chr3:167293827 and chr5:2276656 showed significant hypomethylation (the methylation level of the locus in tumor samples were lower than those in normal samples) among all 13 cancers based on predicted methylation data.**

**Figure S5. Annotations of the enhancer region we found.**

**Table S1. The comparison of prediction performance based on three independent test sets.**

| **GSM3902399** | | | | | | | | | |
| --- | --- | --- | --- | --- | --- | --- | --- | --- | --- |
| **Label** | **Model** | **R** | **RMSE** | **MAE** | **SE** | **SP** | **MCC** | **ACC** | **AUC** |
| β >= 0.5 as 1, β <0.5 as 0 | EAGLING | 0.85 | 0.17 | 0.12 | 0.89 | 0.84 | 0.72 | 0.88 | 0.94 |
|  | Impute knn | 0.88 | 0.24 | 0.19 | 0.96 | 0.89 | 0.86 | 0.94 | 0.94 |
|  | PretiMeth | **0.98** | **0.06** | **0.04** | **0.97** | **0.94** | **0.91** | **0.96** | **0.99** |
| β >= 0.7 as 1, β <=0.3 as 0 | EAGLING | 0.91 | 0.15 | 0.09 | 0.84 | 0.90 | 0.70 | 0.86 | 0.99 |
|  | Impute knn | 0.95 | 0.27 | 0.23 | 0.02 | 0.94 | -0.10 | 0.29 | 0.99 |
|  | PretiMeth | **0.99** | **0.05** | **0.03** | **0.94** | **0.96** | **0.88** | **0.95** | **0.99** |
| **GSM3610355** | | | | | | | | | |
| **Label** | **Model** | **R** | **RMSE** | **MAE** | **SE** | **SP** | **MCC** | **ACC** | **AUC** |
| β >= 0.5 as 1, β <0.5 as 0 | EAGLING | 0.84 | 0.20 | 0.13 | 0.90 | 0.84 | 0.73 | 0.88 | 0.93 |
|  | Impute knn | 0.90 | 0.29 | 0.23 | 0.95 | 0.94 | 0.88 | 0.95 | 0.96 |
|  | PretiMeth | **0.98** | **0.07** | **0.05** | **0.98** | **0.95** | **0.93** | **0.97** | **0.99** |
| β >= 0.7 as 1, β <=0.3 as 0 | EAGLING | 0.87 | 0.19 | 0.12 | 0.81 | 0.83 | 0.62 | 0.82 | 0.96 |
|  | Impute knn | 0.94 | 0.31 | 0.26 | 0.01 | 0.93 | -0.15 | 0.32 | 0.99 |
|  | PretiMeth | **0.99** | **0.06** | **0.04** | **0.96** | **0.95** | **0.91** | **0.96** | **1** |
| **GSM2883349** | | | | | | | | | |
| **Label** | **Model** | **R** | **RMSE** | **MAE** | **SE** | **SP** | **MCC** | **ACC** | **AUC** |
| β >= 0.5 as 1, β <0.5 as 0 | EAGLING | 0.85 | 0.18 | 0.12 | 0.90 | 0.84 | 0.73 | 0.88 | 0.94 |
|  | Impute knn | 0.92 | 0.15 | 0.08 | **0.99** | **0.98** | **0.97** | **0.99** | **0.99** |
|  | PretiMeth | **0.99** | **0.05** | **0.04** | 0.98 | 0.96 | 0.95 | 0.98 | 0.99 |
| β >= 0.7 as 1, β <=0.3 as 0 | EAGLING | 0.89 | 0.16 | 0.10 | 0.82 | 0.86 | 0.64 | 0.83 | 0.97 |
|  | Impute knn | 0.93 | 0.16 | 0.09 | 0.53 | **0.98** | 0.48 | 0.66 | 1 |
|  | PretiMeth | **0.99** | **0.05** | **0.03** | **0.96** | 0.96 | **0.91** | **0.96** | **1** |

**Table S2.** **The prediction performance on WGBS data.**

| **Cell line** | **Model** | **R** | **RMSE** | **MAE** | **SE** | **SP** | **ACC** | **AUC** |
| --- | --- | --- | --- | --- | --- | --- | --- | --- |
| IMR90 | Super high accurate | 0.96 | 0.10 | 0.06 | 0.98 | 0.98 | 0.98 | 0.99 |
|  | Super high accurate  and  High accurate | 0.86 | 0.13 | 0.07 | 0.97 | 0.87 | 0.95 | 0.95 |
| NA12878 | Super high accurate | 0.96 | 0.11 | 0.06 | 0.98 | 0.98 | 0.98 | 0.99 |
|  | Super high accurate  and  High accurate | 0.89 | 0.13 | 0.08 | 0.97 | 0.87 | 0.94 | 0.97 |

**Table S3.** **The number of all analyzed methylation loci and DMLs in each cancer. (**450K: the profiled 450K data; All predictions: the prediction data; Super high & High accurate loci: the more reliable prediction data; DML counts: Number of differential methylated loci.)

| **Cancers** | **450K** | **DML counts** | **All predictions** | **DML counts** | **Super high & High accurate loci** | **DML counts** |
| --- | --- | --- | --- | --- | --- | --- |
| BLCA | 367639 | 113815 | 297738 | 96365 | 132391 | 16128 |
| BRCA | 367620 | 83061 | 297734 | 56170 | 132391 | 7247 |
| COAD | 367639 | 84236 | 297738 | 61343 | 132391 | 8883 |
| ESCA | 367639 | 68269 | 297738 | 45592 | 132391 | 5838 |
| HNSC | 367639 | 87365 | 297738 | 64943 | 132391 | 7938 |
| KIRC | 367639 | 51943 | 297738 | 38184 | 132391 | 2908 |
| KIRP | 367639 | 49274 | 297738 | 30318 | 132391 | 3210 |
| LIHC | 367639 | 109805 | 297738 | 90032 | 132391 | 17592 |
| LUAD | 367639 | 64986 | 297738 | 41328 | 132391 | 4124 |
| LUSC | 367639 | 97483 | 297738 | 68938 | 132391 | 8335 |
| PAAD | 367639 | 41602 | 297738 | 18175 | 132391 | 2768 |
| PRAD | 367639 | 68844 | 297738 | 41056 | 132391 | 6073 |
| THCA | 367639 | 14342 | 297738 | 13061 | 132391 | 980 |

**Table S4.** **The differentially methylated genes related to pan cancers.**

| **Pan-cancer DMGs** | **Cancer counts** |
| --- | --- |
| LOC284933 | 12 |
| BOD1L2、LINC01246、MIR7515HG、MIR7515、ZNF729、ZNF479 | 11 |
| EPS15L1、LOC100506384、MKL1 | 10 |

**Table S5. The number of normal and tumor samples for each cancer.**

| **Cancers** | **Normal samples** | **Tumor samples** |
| --- | --- | --- |
| BLCA | 21 | 419 |
| BRCA | 97 | 797 |
| COAD | 38 | 314 |
| ESCA | 16 | 186 |
| HNSC | 50 | 530 |
| KIRC | 160 | 325 |
| KIRP | 45 | 276 |
| LIHC | 50 | 380 |
| LUAD | 32 | 475 |
| LUSC | 42 | 370 |
| PAAD | 10 | 185 |
| PRAD | 50 | 503 |
| THCA | 56 | 515 |

**
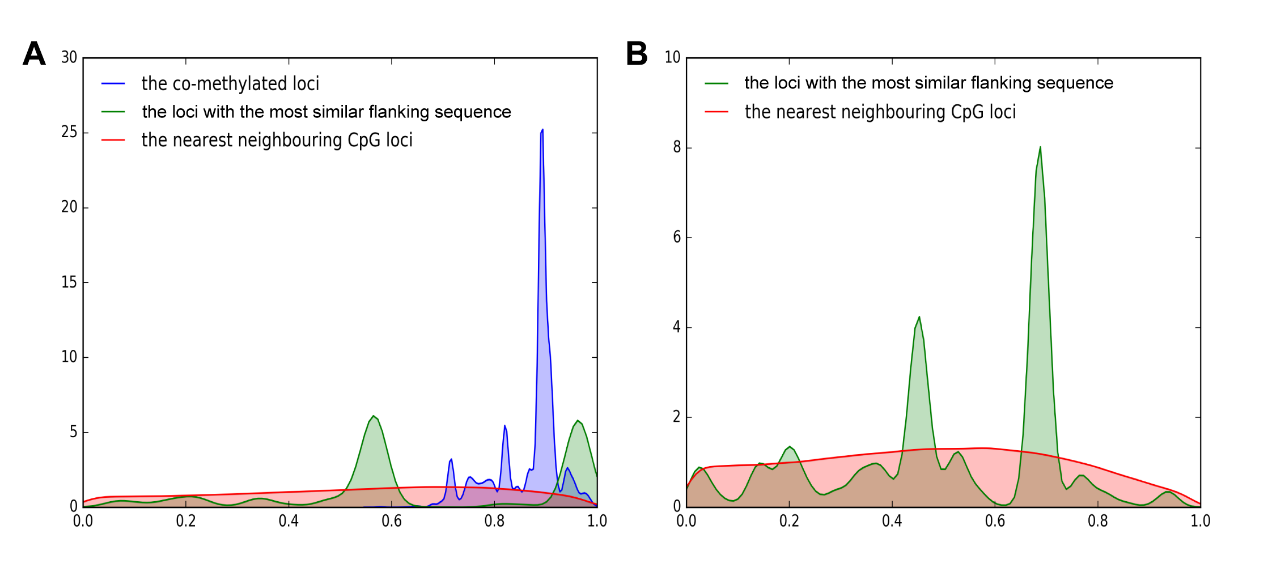
**

**Figure S1. The distribution of correlation coefficients for model loci and the candidate feature loci.** (a) The correlation coefficients with 189,582 model loci limited by the presence of neighbouring loci in 2kb franking coverage. (b) The correlation coefficients with all model loci.


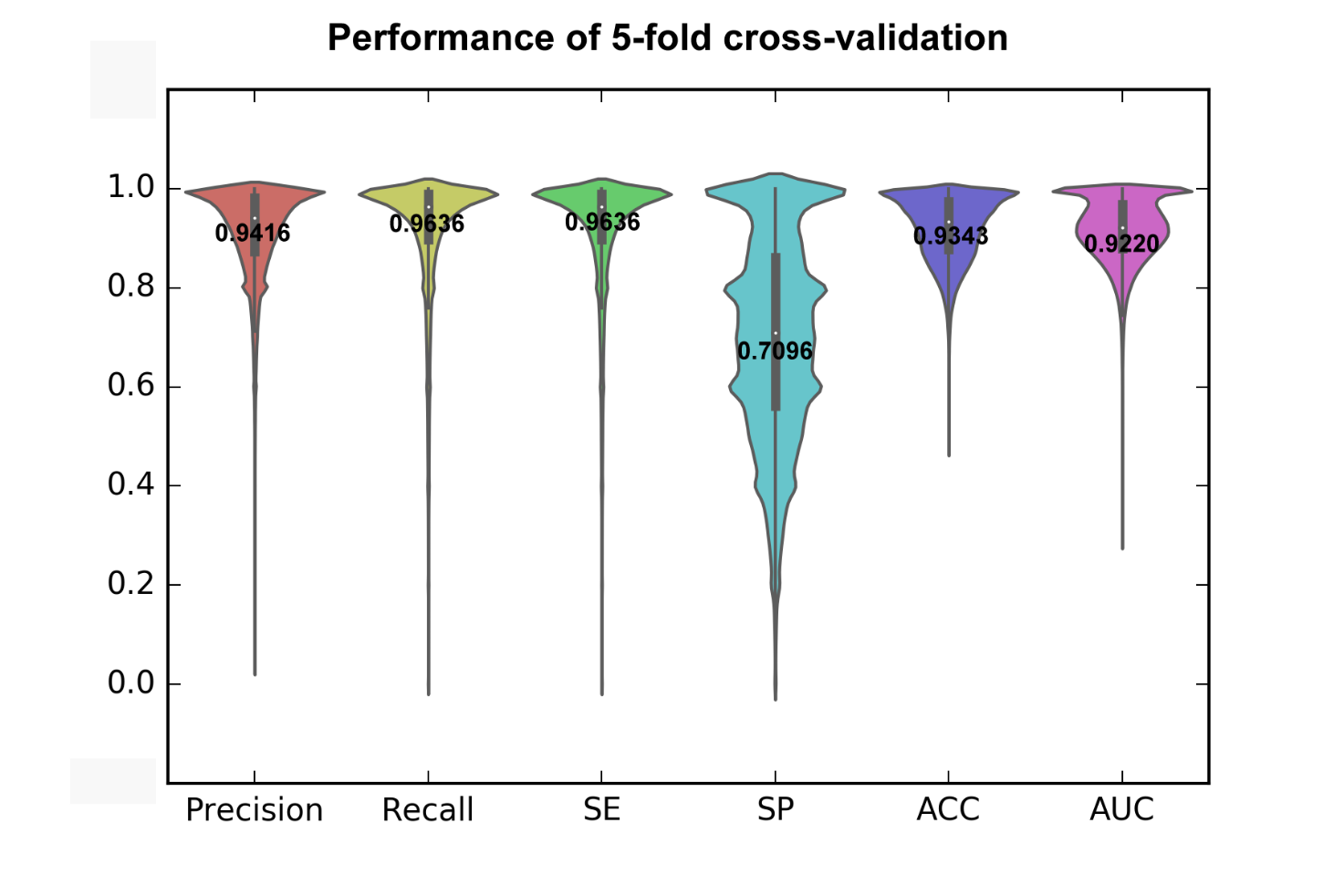


**Figure S2. The classification performance of 413,719 single-locus models on cross-validation.** We defined the methylation value larger than 0.5 as +1, the otherwise as -1, and counted the Precision, Recall, SE, SP, ACC and AUC of the 413,719 single-locus models.


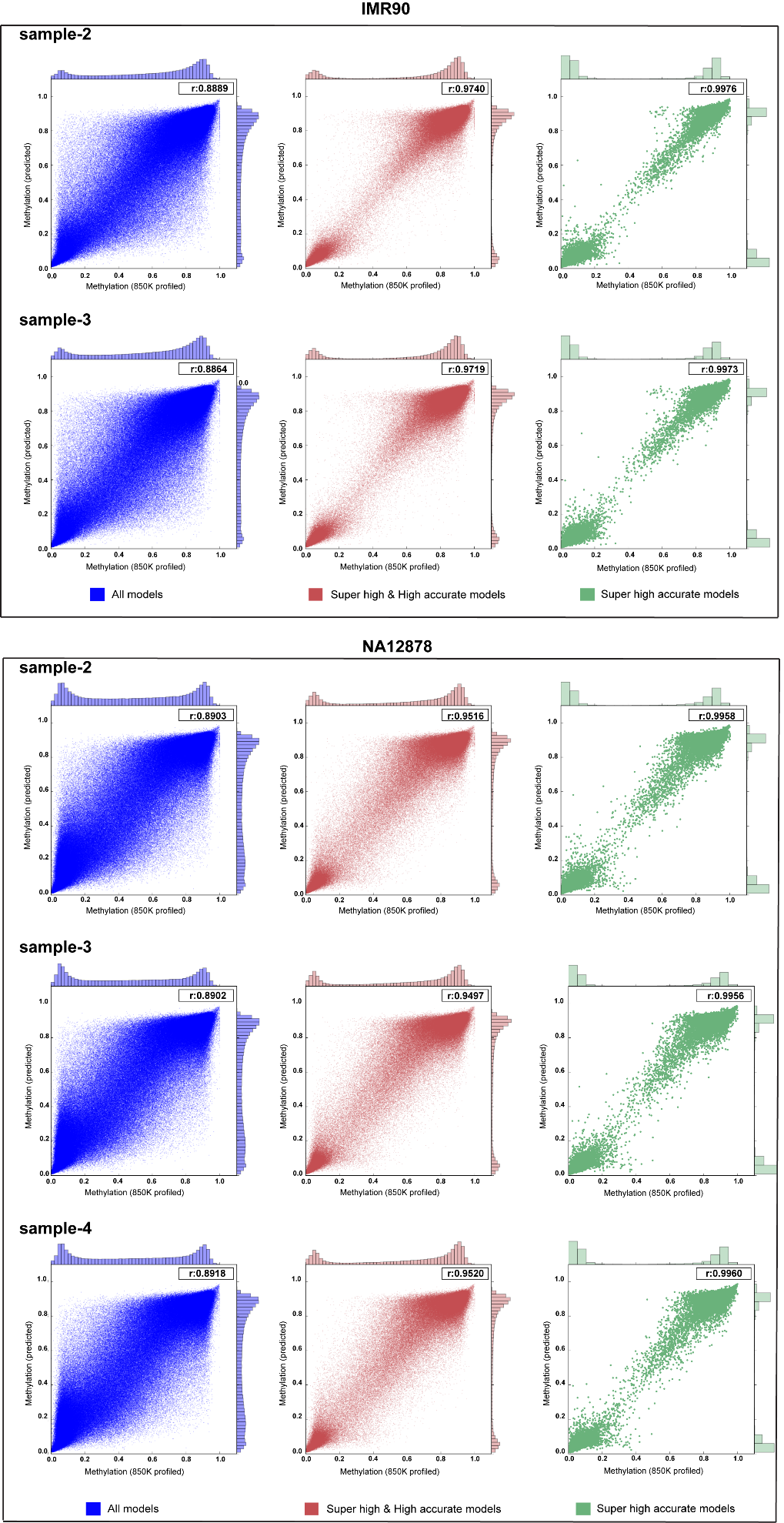


**Figure S3. Scatter plotting the predicted methylation levels and the methylation levels profiled by 850K technology in other samples of IMR90 and NA12878.**


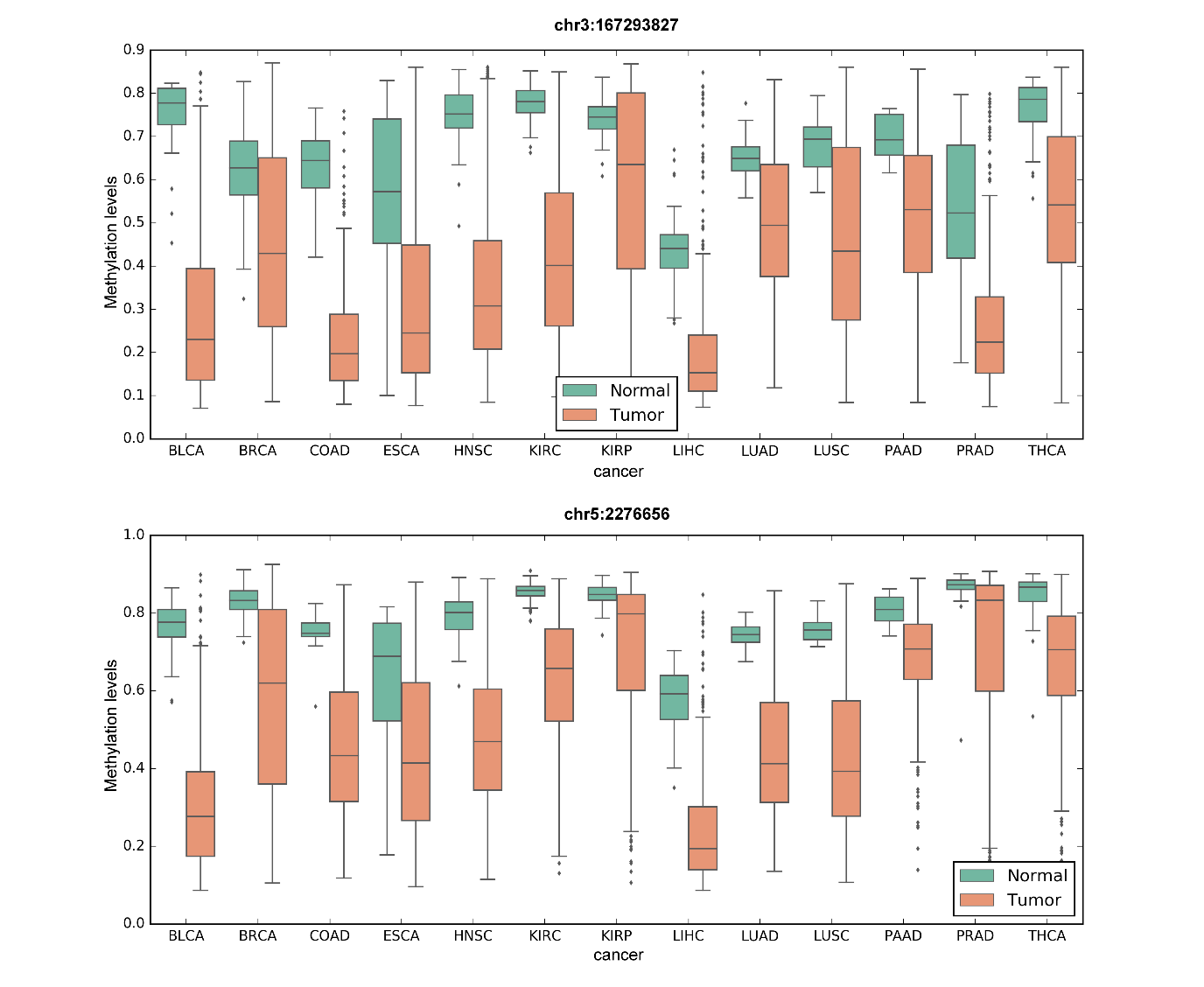


**Figure S4. The probe chr3:167293827 and chr5:2276656 showed significant hypomethylation (the methylation level of the locus in tumor samples were lower than those in normal samples) among all 13 cancers based on predicted methylation data.**


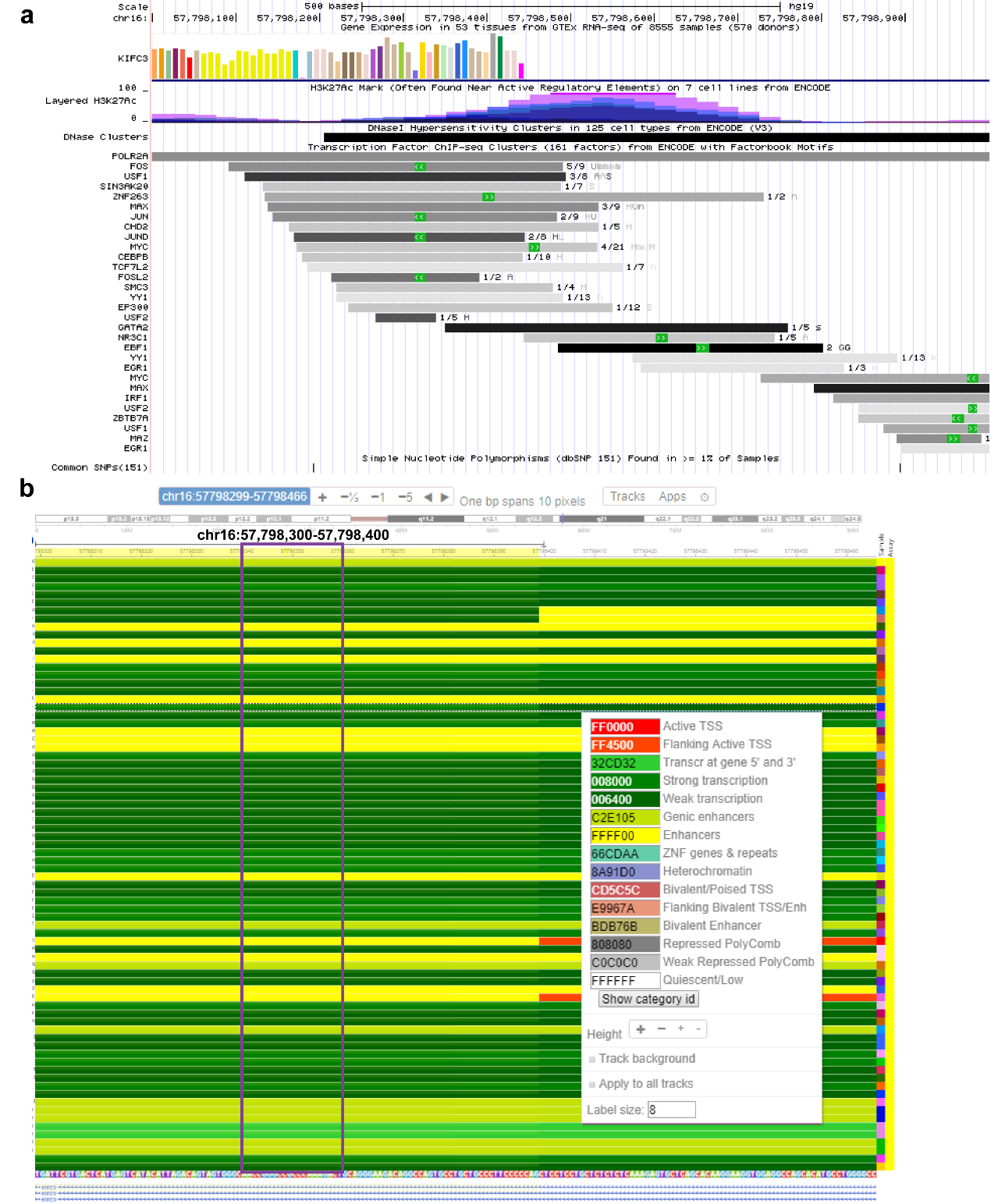


**Figure S5. Annotations of the enhancer region we found.** (a) Illustration of this enhancer region containing the probe chr16:57798350 which is marked by H3K27Ac and bonded several TFs (It is plotted using UCSC genome browser). (b) The enhancer region in roadmap chromatin state by WashU Epigenome Browser (http://epigenomegateway.wustl.edu/browser/), each row represents one primary

somatic cell. The purple box represents the enhancer region chr16: 57798300-

57798400.
